# Rising rates of injection drug use associated infective endocarditis in Virginia with missed opportunities for addiction treatment referral: a retrospective cohort study

**DOI:** 10.1186/s12879-018-3408-y

**Published:** 2018-10-24

**Authors:** Megan E. Gray, Elizabeth T. Rogawski McQuade, W. Michael Scheld, Rebecca A. Dillingham

**Affiliations:** Division of Infectious Diseases and International Health, University of Virginia Health System, PO Box 801379, Charlottesville, Virginia 22908-1391 USA

**Keywords:** Infective endocarditis, Injection drug use, Opioid use disorder

## Abstract

**Background:**

Injection drug use (IDU) is a growing public health threat in Virginia, though there is limited knowledge of related morbidity. The purpose of this study was to describe the temporal, geographic and clinical trends and characteristics of infective endocarditis associated with IDU (IDU-IE) and to identify opportunities for better-quality care of people who inject drugs (PWID).

**Methods:**

We reviewed charts for all admissions coded for both IE and drug use disorders at the University of Virginia Medical Center (UVA) from January 2000 to July 2016. A random sample of 30 admissions coded for IE per year were reviewed to evaluate temporal trends in the proportion of IDU associated IE cases.

**Results:**

There were a total of 76 patients with IDU-IE during the study period, 7.54-fold increase (prevalence ratio: 8.54, 95% CI 3.70–19.72) from 2000 to 2016. The proportion of IE that was IDU-associated increased by nearly 10% each year (prevalence ratio of IDU per year: 1.09, 95% CI: 1.05–1.14). Patients with IDU-IE had longer hospital stays [median days (interquartile range); IDU-IE, 17 (10–29); non-IDU-IE, 10 (6–18); *p*-value = 0.001] with almost twice the cost of admission as those without IDU [median (interquartile range); IDU-IE, $47,899 ($24,578-78,144); non-IDU-IE, $26,460 ($10,220-60,059); *p*-value = 0.001]. In 52% of cases there was no documentation of any discussion regarding addiction treatment.

**Conclusion:**

IDU-IE is a severe infection that leads to significant morbidity and healthcare related costs. IDU-IE rates are increasing and will likely continue to do so without targeted interventions to help PWID. The diagnosis and treatment of IDU-IE provides an opportunity for the delivery of addiction treatment, counseling, and harm reduction strategies.

**Electronic supplementary material:**

The online version of this article (10.1186/s12879-018-3408-y) contains supplementary material, which is available to authorized users.

## Background

Injection drug use (IDU) is a serious public health threat due to the risk for transmission of Human Immunodeficiency Virus (HIV), Hepatitis C Virus (HCV), and overdose related deaths [1, 2]. Bacterial infections caused by IDU are common, the most severe form being infective endocarditis (IE). Though the mortality of IDU has been a major research focus [3], the extent of associated morbidity from other complications, such as IE, has been less extensively characterized.

IDU has increased significantly since the year 2000 in conjunction with a national opioid epidemic, with total opioid overdose related deaths increasing by two-hundred percent in 14 years [3]. This drug epidemic is distinctive in that it primarily affects socioeconomically depressed, rural, and predominately non-Hispanic white populations [4–6]. Sharing injection equipment in social networks of individuals with HIV or HCV infections can lead to viral outbreaks [5, 7]. In addition, using dirty equipment to inject drugs that contain particulate matter and diluents can provoke endothelial damage to heart valves and introduce pathogens into the bloodstream that cause IDU-IE and other localized infections [8].

The incidence of IE in people with IDU is 150–200 per 100,000 person years, approximately 100 times higher than the incidence of IE in the general population [9]. IDU-IE is more likely to affect the right side of the heart and is more frequently caused by Staphylococcal species or polymicrobial infections [9]. Treatment of IE requires long courses of intravenous antibiotics often administered through peripherally inserted central venous catheters. Despite appropriate treatment, recurrence of IE is more common in people who inject drugs (PWID) [10]. The mortality of IDU-IE has been reported to be 10% compared to 20–35% in IE due to other causes (non-IDU-IE) [9]. However, the mortality after valve replacement surgeries is higher in IDU-IE and more than half of those who undergo valve replacement surgeries will require repeated surgical intervention due to persistent injection of drugs [11, 12].

Virginia has one of the fastest growing rates of drug overdose related deaths in the United States [3] and is home to eight of the projected top 5% most vulnerable counties across the United States for viral outbreaks related to IDU [4]. In 2015, emergency department visits for heroin overdose during a nine month period had increased by 89% compared to the same nine month period in 2014, and fatal drug overdoses were the most common cause of unnatural death in 2013 [13]. This led to the declaration of a public health emergency by Virginia’s State Commissioner in October 2016 whereby a statewide standing order was issued that authorized pharmacists to dispense naloxone, an opioid antagonist that reverses the effects of opioids [13]. Several studies have described increasing rates of IDU-IE in the context of increasing IDU [14–18], though no studies have evaluated IDU-IE in Virginia. Few studies have evaluated how IDU is being addressed in the context of a diagnosis of IDU-IE.

Needle and syringe sharing, reuse, and injecting drugs through uncleaned skin are highly implicated in the development of IE, and these practices are common among PWID [19, 20]. Evidence supports the efficacy of several underutilized harm reduction strategies for PWID, such as supervised injection facilities, needle-syringe exchange programs, medication-assisted treatment, and opioid antagonists for overdose treatment [19, 21–26]. Unfortunately, there remains substantial stigma in relation to substance use disorders, which is a barrier to establishing public policies that benefit PWID, such as government funding for abstinence or maintenance-based treatment programs or regulations regarding insurance parity [27]. In order for beneficial policy and social change to take place, more needs to be known about patterns of IDU related morbidity. The purpose of this study was to describe the temporal, geographic and clinical trends and characteristics of IDU-IE in Virginia and to identify opportunities for better-quality care of PWID.

## Methods

### Study design and patient population

A single-center, retrospective cohort study was performed at the University of Virginia Medical Center in Charlottesville, Virginia (UVA). The study site is an 800 bed tertiary care medical center that serves a large rural catchment area extending into West Virginia and far southwest Virginia. The study period ranged from January 1, 2000 to July 1, 2016. Patients were included if they were treated during an inpatient admission at UVA with additional criteria noted below. Patients over the age of 89 were excluded as this age range is considered a patient identifier by the Health Insurance Portability and Accountability Act. Patients under the age of 12 were excluded, similar to other studies evaluating IDU trends [5]. This study was approved by the UVA Institutional Review Board.

The UVA Clinical Data Repository was searched using *International Classification of Diseases, Ninth Revision* and *Tenth Revision* (ICD) diagnosis codes for acute and subacute IE (421, 421.1, 421.9, 424.9, 424.99, 242.91, 112.81, B37.6, I33, I33.0, I33.9, I38, I39). ICD diagnosis codes pertaining to substance abuse, substance abuse counseling and HCV were used to search for patients who inject drugs. Diagnosis codes for cannabinoids were not used. HCV diagnosis codes were used as HCV has often been used as a surrogate marker for IDU [4, 17]. Patient admissions with any of these 453 codes (see Additional file 1) within one year of or at the time of the admission for IE were then selected for chart review. Chart reviews were completed to confirm active IDU and the diagnosis of IE. IDU was conservatively defined as any documentation of injecting drugs within six months of admission for IE. Patients that were suspected of injecting drugs by healthcare staff or family but who denied IDU themselves were not considered to be actively injecting drugs. IE was defined using the modified Duke criteria [28], with inclusion of only definite IE. Patient readmissions for the same episode of IDU-IE related illness were not included.

In order to evaluate the relative trend in total number of cases of IDU-IE compared to other causes of IE, an additional chart review was completed. A stratified random sample of 510 patient admissions for IE, 30 per year, were collected by use of pseudo-random number generator from the 3115 patient admissions ICD coded for IE in the clinical data repository. Admissions with any of the 453 substance use related codes were excluded from the pool used for random sampling. Each chart from the stratified random sample was reviewed to confirm the diagnosis of IE by the modified Duke criteria.

### Data collection

A comprehensive chart review was completed for all patient admissions with IDU-IE in order to collect demographic, clinical and outcomes data. The chart review of the randomly sampled non-IDU-IE patient admissions was limited to verification of IE diagnosis and the causative pathogen. The counties of residence were categorized as rural or urban based on 2010 census data from the Office of Management and Budget [29]. Patients’ health district was also noted.

### Statistical analysis

We compared clinical and demographic characteristics between IDU-IE cases and non-IDU-IE using chi-square and Mann-Whitney tests. We used Poisson regression to model the temporal trend in total IDU-IE admissions over the study period. Year was included in the model as a quadratic variable based on optimal model fit as assessed by Akaike information criteria. We used log-binomial regression to model the temporal trend in the proportion of IDU-IE admissions compared to non-IDU-IE admissions over the study period. All analyses were adjusted for sampling weights.

## Results

### Temporal and geographic trends

**Fig. 1:**
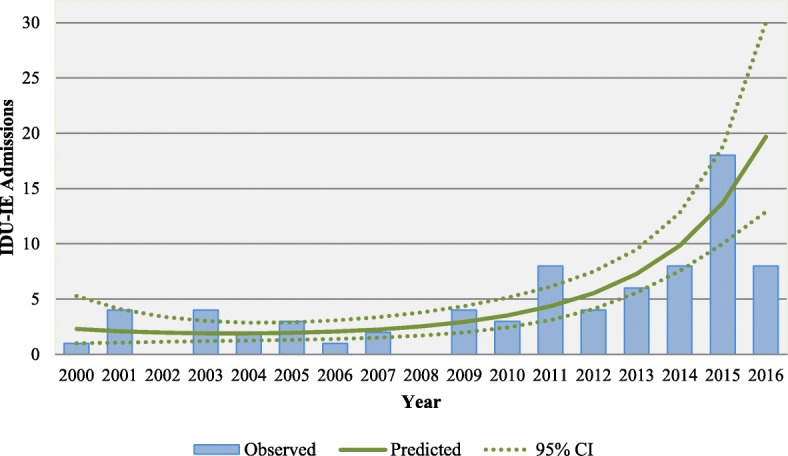
Observed and predicted IDU-IE admissions over time. *In 2016 the observed cases are from only the first 6 months

**Fig. 2:**
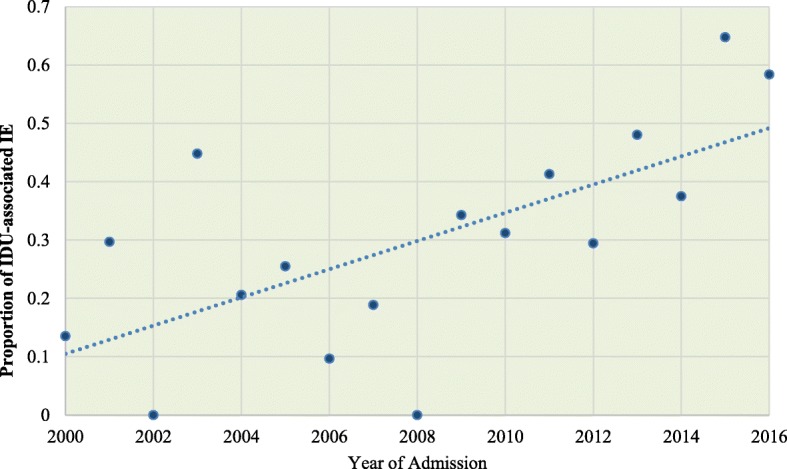
Proportion of IDU-associated IE admissions per year. *Proportions were adjusted for sampling weights

Observed admissions for IDU-IE trended up over time and predictive modeling showed a 7.54-fold increase (prevalence ratio: 8.54, 95% CI 3.70–19.72) from 2000 to 2016 (Fig. 1). Based on this model, 44.6 (95% CI 21–95) IDU-IE admissions would be expected in 2018, which is a 125.8% increase from 2016. The proportion of all IE that was IDU-IE increased by nearly 10% each year (prevalence ratio of IDU-IE per year: 1.09, 95% CI: 1.05–1.14). See Fig. 2.

**Fig. 3:**
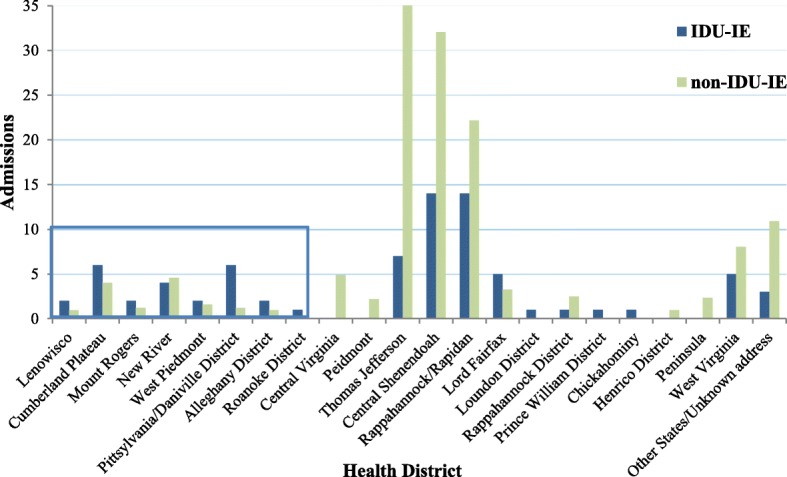
Admissions for IDD-IE and non-IDU-IE by location of residence from January 2000 to July 2016. Blue box is surrounding health districts in Southwest Virginia. Non-IDU-IE cases weighted by year

We estimated that 63 % of all cases of IE in the Southwest region of Virginia were IDU-IE, while 29.4% of the cases were IDU-IE in the remaining regions of Virginia, West Virginia and other states. See Fig. 3.

### IDU-IE and non-IDU-IE

There were a total of 3115 admissions coded for IE from January 2000 to July 2016 at UVA. Of these, 311 admissions also had some type of substance abuse code and these charts were therefore reviewed. A total of 76 admissions were IDU-IE, 235 were excluded for lack of active IDU in the six months prior to admission and/or definitive IE. Of the 510 admissions from the stratified sample of admissions coded for IE, 143 admissions had definite IE. Both populations were predominantly non-Hispanic white race, though patients with IDU-IE were more likely to be non-Hispanic white (96.1% vs 84.7%; *p*-value = 0.02). Patients with IDU-IE were more likely to be younger than non-IDU-IE with a mean age of 35 compared to 61 (p-value < 0.001). See Table 1.

**Table 1:** Characteristics of IDU-IE and non-IDU-IE at UVA from January 2000 to July 2016

Demographic factors	IDU-IE, *N* = 76	Non-IDU-IE *N* = 143	*p*-value
	N (%)	N (%)	
Sex			0.7
Male	86 (60.8)	44 (57.9)	
Female	55 (39.2)	32 (42.1)	
Race			0.02
Caucasian	73 (96.1)	120 (84.7)	
Black	2 (2.6)	21 (14.8)	
Hispanic	1 (1.3)	1 (0.5)	
Mean Age (range)	35 (19–63)	61 (12–89)	< 0.001
Residents of rural counties	24 (31.6)	45 (32.1)	0.9
In-hospital mortality	6 (7.9)	23 (16.6)	0.08
30 day mortality^a^	9 (14.5)	31 (24.4)	0.1
90 day mortality^a^	12 (21.8)	36 (29.3)	0.3
Insurance			< 0.0005
Medicaid	22 (28.9)	11 (7.7)	
Medicare	10 (13.2)	87 (60.8)	
Private	7 (9.2)	28 (19.6)	
Uninsured	34 (44.7)	10 (7)	
Tricare (Federally funded)	0 (0)	1 (0.7)	
State and Local Hospitalization Program	2 (2.6)	2 (1.4)	
Other	1 (1.3)	4 (1.8)	
Pathogen			< 0.0005
MRSA	29 (38.2)	33 (23.5)	
MSSA	17 (22.4)	18 (12.5)	
Other staphylococci	0	7 (4.6)	
*Enteroccus faecalis*	4 (5.3)	19 (13.1)	
Other enterococci	0	5 (3.8)	
Streptococci	7 (9.2)	35 (24.8)	
Candida species	3 (3.9)	6 (4.1)	
Polymicrobial infection	8 (10.5)	0	
Other	4 (5.3)	12 (8.3)	
No pathogen identified	4 (5.3)	7 (5.2)	
	Median (IQR)	Median (IQR)	p-value^b^
Length of stay in days	17 (10–29)	10 (6–18)	0.001
ICU length of stay in days (*n* = 44)^c^	6 (2–12)	5 (2–8)	0.8
Hospital cost in dollars	47,899 (24,578–78,144)	26,460 (10,220–60,059)	0.001

*MSSA* methicillin susceptible *Staphylococcus aureus*, *MRSA* methicillin resistant *Staphylococcus aureus, ICU* intensive care unit

All data were adjusted for sampling weights

^a^Excluding patients with missing mortality data: 14 patients with IDU-IE and 16 patients with non-I DU-IE for 30 day mortality and 21 patients with IDU-IE and 20 with non-IDU-IE for 90 day mortality

^b^*p*-value from Mann-Whitney non-parametric test, other *p*-values from chi-squared test

^c^Excluding 80% of patients with no ICU stay (IDU-IE = 58, non-IDU-IE = 117)

Patients with IDU-IE had longer hospital stays [median days (interquartile range); IDU-IE, 17 (10–29); non-IDU-IE, 10 (6–18); *p*-value = 0.001] with almost twice the cost of admission as those without IDU [median (interquartile range); IDD-IE, $47,899 ($24,578-78,144); non-IDU-IE, $26,460 ($10,220-60,059); *p*-value = 0.001]. Forty-five percent of IDU-IE patients were uninsured and 29% were on Medicaid, while 7% of patients without IDU were uninsured and 7.7% were on Medicaid. Thirty-day and ninety-day mortality data were available for 189 (86%) and 178 (81%) patients respectively; there was no significant difference in mortality between patients with and without IDU.

### Clinical and demographic results for IDU-IE

#### Clinical features and comorbid conditions

Documented fever at the time of admission was present in 36 (47.4%) patients with IDU-IE. Fifteen patients (19.7%) presented with septic shock and five patients (6.6%) presented with severe congestive heart failure. Twenty-four (31.6%) patients had a history of IE. Alcohol use disorder was present in 15 (19.7%) patients. Only five (6.6%) patients were infected with HIV. However, 50 (66%) of patients had been exposed to HCV based on a positive Hepatitis C antibody and negative viral load, with 33 (42.5%) patients having acute or chronic HCV infections with detectable viral loads. Not all patients were screened for HIV or HCV, those without available test results were presumed negative. The majority of IDU-IE patients were injecting some form of opioid (*n* = 51, 67%). See Table 2.

**Table 2:** Clinical characteristics of patients admitted for IDU-IE treatment from January 2000 to July 2016

Clinical Characteristics	N (%)
Right-sided endocarditis	36 (47)
Tricuspid valve	35 (46)
Pulmonic valve	0
Tricuspid and pulmonic valves	1 (1.3)
Left-sided endocarditis	24 (31.6)
Mitral valve	10 (13.2)
Aortic valve	12 (15.8)
Mitral and aortic valves	2 (2.6)
Unknown	2 (2.6)
Mixed (right and left) endocarditis	10 (13.2)
Cardiac device lead	1 (1.3)
No endocardial disease seen	3 (4)
Heart disease history	
Bicuspid aortic valve	1 (1.3)
Congenital heart disease	1 (1.3)
Myxomatous mitral valve	1 (1.3)
Prosthetic valve	11 (14.5)
History of endocarditis	24 (31.6)
Clinical Features	
Fever on presentation	36 (47.4)
Septic shock	15 (19.7)
Severe congestive heart failure	5 (6.6)
Indolent symptoms^a^	56 (73.7)
Indwelling catheter on admission	18 (23.7)
Need for CRRT during admission	12 (15.8)
Co-infections	
Human Immunodeficiency Virus	5 (6.6)
Hepatitis C Virus	
Acute and chronic	33 (42.5)
Past exposure	17 (22.4)
Hepatitis B Virus	
Acute	1 (1.3)
Past exposure	6 (8)
Co-morbid conditions	
Cirrhosis	2 (2.6)
Diabetes	5 (6.6)
End-stage renal disease	1 (1.3)
COPD/Active malignancy	0
Vascular/ Immunologic Phenomenon	
Janeway lesions	7 (9.2)
Splinter hemorrhages	4 (5.3)
Roth spots	2 (2.6)
Osler nodes	6 (8)
Glomerulonephritis	1 (1.3)
Septic pulmonary emboli/infarction	42 (55.3)
Cerebrovascular related events	19 (25)
Emboli to spleen	3 (3.9)
Emboli to bone	4 (5.3)
Septic arthritis	2 (2.6)
Type of injection drug	
Opioids, all^b^	51 (67)
Heroin	22 (29)
Morphine	22 (29)
Hydromorphone	4 (5.3)
Oxymorphone	2 (2.6)
Oxycodone hydrochloride XL	1 (1.3)
Buprenorphine	2 (2.6)
Not specified	16 (21)
Methamphetamines	20 (26.3)
Bath Salts	4 (5.3)
Cocaine	12 (16)
Unknown type	9 (11.2)
Valve surgery performed	31 (41)
Readmissions within 6 months	
One readmission	17 (22.4)
Two readmissions	7 (9.2)
Three readmissions	1 (1.3)

*CRRT* continuous renal replacement therapy, *COPD* chronic obstructive pulmonary disease

^a^Indolent symptoms defined as: fatigue, weight loss, night sweats, reported fevers

^b^Opioid injection type and substance type counts do not add up to total opioid users as some individuals reported injecting several types of substances

#### Substance use disorder treatment and patient disposition

The predominant post-hospital disposition among IDU-IE patients was to home with a home health agency to assist with intravenous antibiotic treatment (*n* = 35, 44.7%). Twenty-six percent of patients went to some type of health care facility including: skilled nursing facilities (*n* = 10), transitional care hospitals (*n* = 4), or acute rehabilitation centers (*n* = 6). Six patients (6.6%) died in the hospital. Cause of death was related to IE in all cases, including one death due to a brain abscess, one death due to an aortic root abscess, and two deaths from septic shock. Three patients (4%) left against medical advice and three patients (4%) were sent back to jail. Seven patients (8%) were able to go home without any intravenous catheter as they completed their treatment in the hospital. All other patients left the hospital with a peripherally inserted central venous catheter or other type of central venous catheter or port.

Thirteen percent of individuals were receiving long-acting opioid agonists (buprenorphine or methadone) for treatment of their substance use disorder at the time of their admission, while 8% had documentation of long-acting opioid agonist treatment in the past. Forty-eight (63%) of individuals had an opioid listed on their discharge medication list. From the first five years to the last five years of our study period this proportion increased from 8/17 (47.1%) to 41/59 (69.5%) (*p*-value = 0.09). However, only 53% of discharge summaries documented IDU or substance use disorder as a problem. In 52% of cases there was no documentation of any discussion regarding substance use disorder treatment or available resources. In 28 (36.8%) patients there was documentation from a social worker regarding resources for substance use disorder being offered to the patient. Six (8%) patients had in-patient consultations from pain management, psychiatry or chronic-pain services in regards to their IDU. One patient had inpatient addiction rehabilitation arranged, but the patient was not able to go. The facility would not allow the patient’s admission with a peripherally inserted central venous catheter. One patient was allowed to leave the hospital to go to Alcoholics Anonymous meetings on furlough during their hospital admission.

## Discussion

A dramatic increase in the number of admissions for IDU-IE was seen at UVA from 2000 to 2016. Individuals with IDU-IE were more likely to be non-Hispanic white race and were younger than those without IDU. Median hospital length of stay was 70% longer and the median hospital cost was nearly two times the cost for those without IDU. A larger percentage of patients IDU-IE were uninsured (55%) compared to patients with non-IDU-IE (7%). Evaluation of the clinical characteristics of IDU-IE found that many patients presented to the hospital acutely ill with high rates of septic shock (19.7%). This is more than double what was seen in a one-year French cohort study (9%) of IE cases [30]. There were additionally high requirements for chronic renal replacement therapy (15.8%). IDU-IE is associated with right heart involvement, our results showed that a significant number of patients 24 (36%) patients actually had left heart involvement. There were also noteworthy embolic complications with septic pulmonary emboli seen in 42 (55.3%) patients and cerebrovascular related events in 19 (25%) patients. IDU-IE did have less in-patient mortality (7.9% IDU-IE, 16.6% non-IDU-IE), however, censored 90-day mortality in those with IDU-IE approached the mortality of the non-IDU-IE group (21.8% IDU-IE vs 29.3% non-IDU-IE, *p*-value = 0.3). The number of patients with previous IE (31.6%) and readmissions (22.4%) highlights the need for further prevention strategies. The high acuity at the time of hospital admission may be affected by delayed patient presentation. This could be partially driven by anticipatory fear of legal repercussions, uninsured status, or concern for withdrawal symptoms.

Increasing rates of IDU-IE in Virginia are consistent with statewide data showing an over 350% increase in rates of acute HCV, which is highly correlated with IDU, during a similar time period [5]. The causes of increasing rates of IDU in Virginia and nationally over this period are at least in part due to increases in opioid prescribing. Prescriptions for opioids have increased nationally from 2007 to 2012 [31] and the southwest region of Virginia prescribes considerably more than the rest of the state [32]. Indeed, in our study the number of patients discharged with an opioid medication on their medication list increased by 22.4%.

IDU-IE and other acute bacterial infections associated with high morbidity, mortality, and costs, may be important metrics to define regions in need of funding for additional addiction treatment and harm reduction services. Policy-makers often allocate public funds for substance use disorder treatment or harm reduction strategies based on rates of HIV and viral hepatitis since there is infrastructure to measure these rates. In our study, known prevalence of HIV (6.6%) and acute and chronic HCV (42.5%) were relatively low. However, increasing rates of IDU-IE may herald potential viral outbreaks, and IDU-IE’s high morbidity and extensive healthcare costs are growing. Tracking of IDU-IE should be considered as an earlier warning sign of unsafe injection practices and the potential for blood-borne viral outbreaks. With this additional surveillance, regions with known increases in IDU-IE or other IDU-related bacterial infections could be targeted as priority areas for the development, authorization, and implementation of evidence-based substance use disorder treatment programs and harm reduction packages. This is especially important to consider in the context of Virginia’s Bill 2317, which allows for syringe service programs as of January 12, 2017 and was passed with a main goal of reducing the transmission of blood borne pathogens [33]. Unfortunately, infrastructure for tracking IDU-IE is not currently available. State level surveillance of IDU-IE could be possible with strategies such as mandatory reporting of inpatient admissions for this condition. National level surveillance could be streamlined with the addition of ICD codes to address IDU and both infectious and non-infectious complications of IDU.

In our study a minority of patients were offered resources for substance use disorder treatment by a social worker or seen by consulting physician teams regarding their IDU. Several factors contribute to these low levels of substance use disorder treatment discussion and initiation. The capacity of available maintenance therapy programs, abstinence therapy programs, and harm reduction strategies do not meet national or the state of Virginia’s demands. In 2014 the rate of opioid dependence in Virginia was 6.5–9.2 per 1000 person years, while the capacity for medication assisted treatment was 0.7–3 per 1000 person years [34]. Some rural areas in the United States have an average two year wait time for medication assisted treatment [35], in part due to insufficient physicians with the required expertise. Many addiction treatment programs are unable to bill insurance and do not receive needed state funding [36]. Deficiencies of available resources and the perception of recidivism by health care providers may make efforts to initiate treatment discussions feel futile. Lastly, stigmatization of IDU and substance use disorders may lead to the perception that the condition represents a moral failing rather than a medical illness [27].

Absence of addiction treatment is not unique to our study site. A similar study evaluating substance use disorder treatment among persons with IDU-IE showed high readmission rates for IDU-related infections, recurrent IDU-IE and high mortality. Only a quarter of patients were offered addiction consultations or psychiatry consultations for IDU [37]. Factors contributing to IDU, such as substance use disorder, must not be overlooked while the complications of IDU are treated in the hospital setting. In addition to enhancing availability of medication assisted treatment and treatment services, including treatment of withdrawal, a multidisciplinary approach with counseling by trained therapists is useful to address underlying factors such as childhood trauma [38]. An inpatient hospitalization is an opportunity to offer these services, link patients to care, and to offer harm reduction strategies. Specifically, education on safe injecting practices, the prescribing of naloxone to empower individuals to treat unintentional overdoses and the prescribing of HIV pre-exposure prophylaxis with adjunct HIV education and counseling [37, 39]. Relationships between healthcare staff and patients with IDU may be challenging due to many factors, not limited to real and perceived stigma [40]. Concerted efforts to better educate healthcare workers and the community regarding IDU-associated substance use disorders as curable diseases may reduce stigma and improve the care of PWID both inside and outside of the hospital setting [41].

The median 17 day hospital stay and six week intravenous antibiotic treatment course required for each case of IDU-IE is an additional opportunity for multidisciplinary addiction treatment. Almost half of all IDU-IE patients were discharged from the hospital with home health agencies and an additional quarter of patients were sent to some type of nursing facility. Residential addiction treatment services that offer antibiotic infusions for IE treatment have been shown to be cost effective in reducing hospital length of stay. There are concerns related to sending PWID home with peripherally inserted central catheters, largely related to risk for catheter infections from catheter misuse. In rural areas, this is often the only option due to lack of insurance and/or lack of facilities near patients’ residence. Therefore, in these settings, engaging home health agencies to assist in providing addiction treatment services in conjunction with antibiotic infusions could be helpful [21].

This study was limited by potential errors associated with coding, specifically the lack of an ICD code for IDU. The chart review process was done in part to account for these errors. The conservative criteria use to define both IDU and IE may have led to missed cases of IDU-IE. The study did not determine the total number of admissions for IE, therefore the true proportion of IE due to IDU could not be determined. Finally, our institution implemented a new electronic medical record in 2011, which resulted in some changes in documentation practices.

## Conclusion

IDU-IE is a severe infection that leads to significant morbidity and healthcare related costs. IDU-IE rates are increasing and will likely continue to do so without targeted interventions to help PWID. The diagnosis and treatment of IDU-IE provides an opportunity for the delivery of addiction treatment, counseling, and harm reduction strategies.

## Summary

Numbers of infective endocarditis cases related to injection drug use (IDU) have increased significantly in Virginia. While infective endocarditis is treated medically, opportunities for addiction treatment referral are missed.

## Additional file

Additional file 1: ICD diagnosis codes pertaining to substance abuse, substance abuse counseling and HCV. (PDF 228 kb)

## Abbreviations

HCV: Hepatitis C Virus
HIV: human immunodeficiency virus
ICD: International Classification of Diseases
IDU: injection drug use
IDU-IE: injection drug use associated infective endocarditis
IE: infective endocarditis
non-IDU-IE: infective endocarditis not related to injection drug use
PWID: people who inject drugs
UVA: University of Virginia
