# Supplementary material for: Rising rates of injection drug use associated infective endocarditis in Virginia with missed opportunities for addiction treatment referral: a retrospective cohort study
Source: BMC Infect Dis. 2018 Oct 24;18:532. doi: 10.1186/s12879-018-3408-y (PMC6201507; doi:10.1186/s12879-018-3408-y)
Supplement: Supplementary file 1 — ICD diagnosis codes pertaining to substance abuse, substance abuse counseling and HCV. (PDF 228 kb) [file 12879_2018_3408_MOESM1_ESM.pdf]

Additional File 1. ICD diagnosis codes pertaining to substance abuse, substance abuse counseling and HCV

ICD-9 and ICD-10 diagnosis codes pertaining to substance abuse, substance abuse counseling and HCV
V02.6 - VIRAL HEPATITIS CARRIER<br>V02.60 - VIRAL HEPATITIS CARRIER UNSPEC<br>Z71.5 - DRUG ABUSE COUNSELING AND SURVEILLANCE<br>070.7 - VIRAL HEPATITIS C NOS<br>B19.2 - UNSPECIFIED VIRAL HEPATITIS C<br>B19.21 - UNSPECIFIED VIRAL HEPATITIS C WITH HEPATIC COMA<br>B19.20 - UNSPECIFIED VIRAL HEPATITIS C WITHOUT HEPATIC COMA<br>V02.69 - OT VIRAL HEPATITIS CARRIER<br>B18.2 - CHRONIC VIRAL HEPATITIS C<br>V02.62 - HEPATITIS C CARRIER<br>B17.10 - ACUTE HEPATITIS C WITHOUT HEPATIC COMA<br>Z22.52 - CARRIER OF VIRAL HEPATITIS C<br>B17.1 - ACUTE HEPATITIS C<br>B17.11 - ACUTE HEPATITIS C WITH HEPATIC COMA<br>F16.29 - HALLUCINOGEN DEPENDENCE W UNSP HALLUCINOGEN-INDUCED DISORDER<br>F16.283 - HALLUCIGN DEPEND W HALLUCIGN PERSISTING PERCEPTION DISORDER<br>F16.288 - HALLUCINOGEN DEPENDENCE W OTH HALLUCINOGEN-INDUCED DISORDER<br>F16.280 - HALLUCINOGEN DEPENDENCE W ANXIETY DISORDER<br>F16.28 - HALLUCINOGEN DEPENDENCE W OTH HALLUCINOGEN-INDUCED DISORDER<br>F16.250 - HALLUCINOGEN DEPENDENCE W PSYCHOTIC DISORDER W DELUSIONS<br>F16.259 - HALLUCINOGEN DEPENDENCE W PSYCHOTIC DISORDER, UNSP<br>F16.251 - HALLUCINOGEN DEPENDENCE W PSYCHOTIC DISORDER W HALLUCIN<br>F16.25 - HALLUCINOGEN DEPENDENCE W PSYCHOTIC DISORDER<br>F16.24 - HALLUCINOGEN DEPENDENCE W HALLUCINOGEN-INDUCED MOOD DISORDER<br>F16.229 - HALLUCINOGEN DEPENDENCE WITH INTOXICATION, UNSPECIFIED<br>F16.221 - HALLUCINOGEN DEPENDENCE WITH INTOXICATION WITH DELIRIUM<br>F16.220 - HALLUCINOGEN DEPENDENCE WITH INTOXICATION, UNCOMPLICATED<br>F16.22 - HALLUCINOGEN DEPENDENCE WITH INTOXICATION<br>F16.21 - HALLUCINOGEN DEPENDENCE, IN REMISSION<br>F16.20 - HALLUCINOGEN DEPENDENCE, UNCOMPLICATED<br>F16.188 - HALLUCINOGEN ABUSE WITH OTHER HALLUCINOGEN-INDUCED DISORDER<br>F16.19 - HALLUCINOGEN ABUSE WITH UNSP HALLUCINOGEN-INDUCED DISORDER<br>F16.2 - HALLUCINOGEN DEPENDENCE<br>F16.150 - HALLUCINOGEN ABUSE W PSYCHOTIC DISORDER W DELUSIONS<br>F16.18 - HALLUCINOGEN ABUSE WITH OTHER HALLUCINOGEN-INDUCED DISORDER<br>F16.159 - HALLUCINOGEN ABUSE W PSYCHOTIC DISORDER, UNSP<br>F16.180 - HALLUCINOGEN ABUSE W HALLUCINOGEN-INDUCED ANXIETY DISORDER

F16.183 - HALLUCIGN ABUSE W HALLUCIGN PERSISTING PERCEPTION DISORDER
F16.151 - HALLUCINOGEN ABUSE W PSYCHOTIC DISORDER W HALLUCINATIONS
F16.15 - HALLUCINOGEN ABUSE W HALLUCINOGEN-INDUCED PSYCHOTIC DISORDER
F16.14 - HALLUCINOGEN ABUSE WITH HALLUCINOGEN-INDUCED MOOD DISORDER
F16.122 - HALLUCINOGEN ABUSE W INTOXICATION W PERCEPTUAL DISTURBANCE
F16.129 - HALLUCINOGEN ABUSE WITH INTOXICATION, UNSPECIFIED
F16.121 - HALLUCINOGEN ABUSE WITH INTOXICATION WITH DELIRIUM
F16.120 - HALLUCINOGEN ABUSE WITH INTOXICATION, UNCOMPLICATED
F16.12 - HALLUCINOGEN ABUSE WITH INTOXICATION
F16.1 - HALLUCINOGEN ABUSE
F15.988 - OTH STIMULANT USE, UNSP WITH OTH STIMULANT-INDUCED DISORDER
F15.99 - OTH STIMULANT USE, UNSP WITH UNSP STIMULANT-INDUCED DISORDER
F16.10 - HALLUCINOGEN ABUSE, UNCOMPLICATED
F15.982 - OTH STIMULANT USE, UNSP W STIMULANT-INDUCED SLEEP DISORDER
F15.981 - OTH STIMULANT USE, UNSP W STIM-INDUCE SEXUAL DYSFUNCTION
F15.98 - OTH STIMULANT USE, UNSP WITH OTH STIMULANT-INDUCED DISORDER
F15.959 - OTH STIMULANT USE, UNSP W STIM-INDUCE PSYCH DISORDER, UNSP
F15.980 - OTH STIMULANT USE, UNSP W STIMULANT-INDUCED ANXIETY DISORDER
F15.951 - OTH STIM USE, UNSP W STIM-INDUCE PSYCH DISORDER W HALLUCIN
F15.950 - OTH STIM USE, UNSP W STIM-INDUCE PSYCH DISORDER W DELUSIONS
F15.93 - OTHER STIMULANT USE, UNSPECIFIED WITH WITHDRAWAL
F15.94 - OTH STIMULANT USE, UNSP WITH STIMULANT-INDUCED MOOD DISORDER
F15.95 - OTH STIMULANT USE, UNSP W STIM-INDUCE PSYCHOTIC DISORDER
F15.922 - OTH STIMULANT USE, UNSP W INTOX W PERCEPTUAL DISTURBANCE
F15.929 - OTHER STIMULANT USE, UNSP WITH INTOXICATION, UNSPECIFIED
F15.921 - OTHER STIMULANT USE, UNSPECIFIED WITH INTOXICATION DELIRIUM
F15.920 - OTHER STIMULANT USE, UNSP WITH INTOXICATION, UNCOMPLICATED
F15.9 - OTHER STIMULANT USE, UNSPECIFIED
F15.92 - OTHER STIMULANT USE, UNSPECIFIED WITH INTOXICATION
F15.90 - OTHER STIMULANT USE, UNSPECIFIED, UNCOMPLICATED
F15.29 - OTH STIMULANT DEPENDENCE W UNSP STIMULANT-INDUCED DISORDER
F15.282 - OTH STIMULANT DEPENDENCE W STIMULANT-INDUCED SLEEP DISORDER
F15.288 - OTH STIMULANT DEPENDENCE WITH OTH STIMULANT-INDUCED DISORDER
F15.281 - OTH STIMULANT DEPENDENCE W STIM-INDUCE SEXUAL DYSFUNCTION
F15.280 - OTH STIMULANT DEPENDENCE W STIM-INDUCE ANXIETY DISORDER
F15.28 - OTH STIMULANT DEPENDENCE WITH OTH STIMULANT-INDUCED DISORDER
F15.251 - OTH STIMULANT DEPEND W STIM-INDUCE PSYCH DISORDER W HALLUCIN
F15.259 - OTH STIMULANT DEPEND W STIM-INDUCE PSYCHOTIC DISORDER, UNSP
F15.25 - OTH STIMULANT DEPENDENCE W STIM-INDUCE PSYCHOTIC DISORDER
F15.250 - OTH STIM DEPEND W STIM-INDUCE PSYCH DISORDER W DELUSIONS
F15.24 - OTH STIMULANT DEPENDENCE W STIMULANT-INDUCED MOOD DISORDER

F15.23 - OTHER STIMULANT DEPENDENCE WITH WITHDRAWAL
F15.229 - OTHER STIMULANT DEPENDENCE WITH INTOXICATION, UNSPECIFIED
F15.222 - OTH STIMULANT DEPENDENCE W INTOX W PERCEPTUAL DISTURBANCE
F15.221 - OTHER STIMULANT DEPENDENCE WITH INTOXICATION DELIRIUM
F15.220 - OTHER STIMULANT DEPENDENCE WITH INTOXICATION, UNCOMPLICATED
F15.22 - OTHER STIMULANT DEPENDENCE WITH INTOXICATION
F15.21 - OTHER STIMULANT DEPENDENCE, IN REMISSION
F15.20 - OTHER STIMULANT DEPENDENCE, UNCOMPLICATED
F15.2 - OTHER STIMULANT DEPENDENCE
F15.19 - OTHER STIMULANT ABUSE WITH UNSP STIMULANT-INDUCED DISORDER
F15.182 - OTHER STIMULANT ABUSE WITH STIMULANT-INDUCED SLEEP DISORDER
F15.188 - OTHER STIMULANT ABUSE WITH OTHER STIMULANT-INDUCED DISORDER
F15.181 - OTH STIMULANT ABUSE W STIMULANT-INDUCED SEXUAL DYSFUNCTION
F15.180 - OTH STIMULANT ABUSE WITH STIMULANT-INDUCED ANXIETY DISORDER
F15.159 - OTH STIMULANT ABUSE W STIM-INDUCE PSYCHOTIC DISORDER, UNSP
F15.18 - OTHER STIMULANT ABUSE WITH OTHER STIMULANT-INDUCED DISORDER
F15.151 - OTH STIMULANT ABUSE W STIM-INDUCE PSYCH DISORDER W HALLUCIN
F15.150 - OTH STIMULANT ABUSE W STIM-INDUCE PSYCH DISORDER W DELUSIONS
F15.15 - OTH STIMULANT ABUSE W STIMULANT-INDUCED PSYCHOTIC DISORDER
F15.14 - OTHER STIMULANT ABUSE WITH STIMULANT-INDUCED MOOD DISORDER
F15.129 - OTHER STIMULANT ABUSE WITH INTOXICATION, UNSPECIFIED
F15.122 - OTH STIMULANT ABUSE W INTOXICATION W PERCEPTUAL DISTURBANCE
F15.121 - OTHER STIMULANT ABUSE WITH INTOXICATION DELIRIUM
F15.120 - OTHER STIMULANT ABUSE WITH INTOXICATION, UNCOMPLICATED
F15.12 - OTHER STIMULANT ABUSE WITH INTOXICATION
F15.10 - OTHER STIMULANT ABUSE, UNCOMPLICATED
F15.1 - OTHER STIMULANT ABUSE
F14.99 - COCAINE USE, UNSP WITH UNSPECIFIED COCAINE-INDUCED DISORDER
F14.988 - COCAINE USE, UNSPECIFIED WITH OTHER COCAINE-INDUCED DISORDER
F14.982 - COCAINE USE, UNSPECIFIED WITH COCAINE-INDUCED SLEEP DISORDER
F14.981 - COCAINE USE, UNSP WITH COCAINE-INDUCED SEXUAL DYSFUNCTION
F14.980 - COCAINE USE, UNSP WITH COCAINE-INDUCED ANXIETY DISORDER
F14.98 - COCAINE USE, UNSPECIFIED WITH OTH COCAINE-INDUCED DISORDER
F14.959 - COCAINE USE, UNSP W COCAINE-INDUCED PSYCHOTIC DISORDER, UNSP
F14.951 - COCAINE USE, UNSP W COCAINE-INDUC PSYCH DISORDER W HALLUCIN
F14.950 - COCAINE USE, UNSP W COCAINE-INDUC PSYCH DISORDER W DELUSIONS
F14.95 - COCAINE USE, UNSP WITH COCAINE-INDUCED PSYCHOTIC DISORDER
F14.94 - COCAINE USE, UNSPECIFIED WITH COCAINE-INDUCED MOOD DISORDER
F14.929 - COCAINE USE, UNSPECIFIED WITH INTOXICATION, UNSPECIFIED
F14.922 - COCAINE USE, UNSP W INTOXICATION WITH PERCEPTUAL DISTURBANCE
F14.921 - COCAINE USE, UNSPECIFIED WITH INTOXICATION DELIRIUM

F14.920 - COCAINE USE, UNSPECIFIED WITH INTOXICATION, UNCOMPLICATED
F14.92 - COCAINE USE, UNSPECIFIED WITH INTOXICATION
F14.90 - COCAINE USE, UNSPECIFIED, UNCOMPLICATED
F14.9 - COCAINE USE, UNSPECIFIED
F14.29 - COCAINE DEPENDENCE WITH UNSPECIFIED COCAINE-INDUCED DISORDER
F14.288 - COCAINE DEPENDENCE WITH OTHER COCAINE-INDUCED DISORDER
F14.282 - COCAINE DEPENDENCE WITH COCAINE-INDUCED SLEEP DISORDER
F14.281 - COCAINE DEPENDENCE WITH COCAINE-INDUCED SEXUAL DYSFUNCTION
F14.280 - COCAINE DEPENDENCE WITH COCAINE-INDUCED ANXIETY DISORDER
F14.28 - COCAINE DEPENDENCE WITH OTHER COCAINE-INDUCED DISORDER
F14.259 - COCAINE DEPENDENCE W COCAINE-INDUC PSYCHOTIC DISORDER, UNSP
F14.251 - COCAINE DEPEND W COCAINE-INDUC PSYCHOTIC DISORDER W HALLUCIN
F14.250 - COCAINE DEPEND W COCAINE-INDUC PSYCH DISORDER W DELUSIONS
F14.25 - COCAINE DEPENDENCE WITH COCAINE-INDUCED PSYCHOTIC DISORDER
F14.24 - COCAINE DEPENDENCE WITH COCAINE-INDUCED MOOD DISORDER
F14.23 - COCAINE DEPENDENCE WITH WITHDRAWAL
F14.229 - COCAINE DEPENDENCE WITH INTOXICATION, UNSPECIFIED
F14.222 - COCAINE DEPENDENCE W INTOXICATION W PERCEPTUAL DISTURBANCE
F14.221 - COCAINE DEPENDENCE WITH INTOXICATION DELIRIUM
F14.220 - COCAINE DEPENDENCE WITH INTOXICATION, UNCOMPLICATED
F14.22 - COCAINE DEPENDENCE WITH INTOXICATION
F14.2 - COCAINE DEPENDENCE
F14.20 - COCAINE DEPENDENCE, UNCOMPLICATED
F14.21 - COCAINE DEPENDENCE, IN REMISSION
F14.188 - COCAINE ABUSE WITH OTHER COCAINE-INDUCED DISORDER
F14.19 - COCAINE ABUSE WITH UNSPECIFIED COCAINE-INDUCED DISORDER
F14.182 - COCAINE ABUSE WITH COCAINE-INDUCED SLEEP DISORDER
F14.181 - COCAINE ABUSE WITH COCAINE-INDUCED SEXUAL DYSFUNCTION
F14.180 - COCAINE ABUSE WITH COCAINE-INDUCED ANXIETY DISORDER
F14.18 - COCAINE ABUSE WITH OTHER COCAINE-INDUCED DISORDER
F14.159 - COCAINE ABUSE WITH COCAINE-INDUCED PSYCHOTIC DISORDER, UNSP
F14.151 - COCAINE ABUSE W COCAINE-INDUC PSYCHOTIC DISORDER W HALLUCIN
F14.150 - COCAINE ABUSE W COCAINE-INDUC PSYCHOTIC DISORDER W DELUSIONS
F14.15 - COCAINE ABUSE WITH COCAINE-INDUCED PSYCHOTIC DISORDER
F14.14 - COCAINE ABUSE WITH COCAINE-INDUCED MOOD DISORDER
F14.129 - COCAINE ABUSE WITH INTOXICATION, UNSPECIFIED
F14.122 - COCAINE ABUSE WITH INTOXICATION WITH PERCEPTUAL DISTURBANCE
F14.121 - COCAINE ABUSE WITH INTOXICATION WITH DELIRIUM
F14.120 - COCAINE ABUSE WITH INTOXICATION, UNCOMPLICATED
F14.10 - COCAINE ABUSE, UNCOMPLICATED
F14.12 - COCAINE ABUSE WITH INTOXICATION

F14.1 - COCAINE ABUSE
F13.99 - SEDATIVE, HYPNOTIC OR ANXIOLYTIC USE, UNSP W UNSP DISORDER
F13.982 - SEDATIVE, HYPNOTIC OR ANXIOLYTIC USE, UNSP W SLEEP DISORDER
F13.981 - SEDATV/HYP/ANXIOLYTC USE, UNSP W SEXUAL DYSFUNCTION
F13.988 - SEDATIVE, HYPNOTIC OR ANXIOLYTIC USE, UNSP W OTH DISORDER
F13.980 - SEDATV/HYP/ANXIOLYTC USE, UNSP W ANXIETY DISORDER
F13.98 - SEDATIVE, HYPNOTIC OR ANXIOLYTIC USE, UNSP W OTH DISORDERS
F13.97 - SEDATV/HYP/ANXIOLYTC USE, UNSP W PERSISTING DEMENTIA
F13.96 - SEDATV/HYP/ANXIOLYTC USE, UNSP W PERSIST AMNESTIC DISORDER
F13.959 - SEDATV/HYP/ANXIOLYTC USE, UNSP W PSYCHOTIC DISORDER, UNSP
F13.951 - SEDATV/HYP/ANXIOLYTC USE, UNSP W PSYCH DISORDER W HALLUCIN
F13.95 - SEDATV/HYP/ANXIOLYTC USE, UNSP W PSYCHOTIC DISORDER
F13.950 - SEDATV/HYP/ANXIOLYTC USE, UNSP W PSYCH DISORDER W DELUSIONS
F13.94 - SEDATIVE, HYPNOTIC OR ANXIOLYTIC USE, UNSP W MOOD DISORDER
F13.931 - SEDATV/HYP/ANXIOLYTC USE, UNSP W WITHDRAWAL DELIRIUM
F13.932 - SEDATV/HYP/ANXIOLYTC USE, UNSP W W/DRAWAL W PERCEPTL DISTURB
F13.939 - SEDATV/HYP/ANXIOLYTC USE, UNSP W WITHDRAWAL, UNSP
F13.930 - SEDATV/HYP/ANXIOLYTC USE, UNSP W WITHDRAWAL, UNCOMPLICATED
F13.93 - SEDATIVE, HYPNOTIC OR ANXIOLYTIC USE, UNSP WITH WITHDRAWAL
F13.921 - SEDATV/HYP/ANXIOLYTC USE, UNSP W INTOXICATION DELIRIUM
F13.929 - SEDATV/HYP/ANXIOLYTC USE, UNSP W INTOXICATION, UNSP
F13.920 - SEDATV/HYP/ANXIOLYTC USE, UNSP W INTOXICATION, UNCOMPLICATED
F13.92 - SEDATIVE, HYPNOTIC OR ANXIOLYTIC USE, UNSP WITH INTOXICATION
F13.9 - SEDATIVE, HYPNOTIC OR ANXIOLYTIC-RELATED USE, UNSPECIFIED
F13.90 - SEDATIVE, HYPNOTIC, OR ANXIOLYTIC USE, UNSP, UNCOMPLICATED
F13.29 - SEDATIVE, HYPNOTIC OR ANXIOLYTIC DEPENDENCE W UNSP DISORDER
F13.288 - SEDATIVE, HYPNOTIC OR ANXIOLYTIC DEPENDENCE W OTH DISORDER
F13.282 - SEDATIVE, HYPNOTIC OR ANXIOLYTIC DEPENDENCE W SLEEP DISORDER
F13.281 - SEDATV/HYP/ANXIOLYTC DEPENDENCE W SEXUAL DYSFUNCTION
F13.28 - SEDATIVE, HYPNOTIC OR ANXIOLYTIC DEPENDENCE W OTH DISORDERS
F13.280 - SEDATV/HYP/ANXIOLYTC DEPENDENCE W ANXIETY DISORDER
F13.26 - SEDATV/HYP/ANXIOLYTC DEPEND W PERSISTING AMNESTIC DISORDER
F13.27 - SEDATV/HYP/ANXIOLYTC DEPENDENCE W PERSISTING DEMENTIA
F13.259 - SEDATV/HYP/ANXIOLYTC DEPENDENCE W PSYCHOTIC DISORDER, UNSP
F13.250 - SEDATV/HYP/ANXIOLYTC DEPEND W PSYCHOTIC DISORDER W DELUSIONS
F13.251 - SEDATV/HYP/ANXIOLYTC DEPEND W PSYCHOTIC DISORDER W HALLUCIN
F13.25 - SEDATV/HYP/ANXIOLYTC DEPENDENCE W PSYCHOTIC DISORDER
F13.24 - SEDATIVE, HYPNOTIC OR ANXIOLYTIC DEPENDENCE W MOOD DISORDER
F13.239 - SEDATV/HYP/ANXIOLYTC DEPENDENCE W WITHDRAWAL, UNSP
F13.231 - SEDATV/HYP/ANXIOLYTC DEPENDENCE W WITHDRAWAL DELIRIUM
F13.232 - SEDATV/HYP/ANXIOLYTC DEPEND W W/DRAWAL W PERCEPTUAL DISTURB

F13.230 - SEDATV/HYP/ANXIOLYTC DEPENDENCE W WITHDRAWAL, UNCOMPLICATED
F13.23 - SEDATIVE, HYPNOTIC OR ANXIOLYTIC DEPENDENCE WITH WITHDRAWAL
F13.229 - SEDATV/HYP/ANXIOLYTC DEPENDENCE W INTOXICATION, UNSP
F13.220 - SEDATV/HYP/ANXIOLYTC DEPENDENCE W INTOXICATION, UNCOMP
F13.221 - SEDATV/HYP/ANXIOLYTC DEPENDENCE W INTOXICATION DELIRIUM
F13.22 - SEDATIVE, HYPNOTIC OR ANXIOLYTIC DEPENDENCE W INTOXICATION
F13.21 - SEDATIVE, HYPNOTIC OR ANXIOLYTIC DEPENDENCE, IN REMISSION
F13.19 - SEDATIVE, HYPNOTIC OR ANXIOLYTIC ABUSE W UNSP DISORDER
F13.20 - SEDATIVE, HYPNOTIC OR ANXIOLYTIC DEPENDENCE, UNCOMPLICATED
F13.188 - SEDATIVE, HYPNOTIC OR ANXIOLYTIC ABUSE W OTH DISORDER
F13.2 - SEDATIVE, HYPNOTIC OR ANXIOLYTIC-RELATED DEPENDENCE
F13.182 - SEDATIVE, HYPNOTIC OR ANXIOLYTIC ABUSE W SLEEP DISORDER
F13.181 - SEDATIVE, HYPNOTIC OR ANXIOLYTIC ABUSE W SEXUAL DYSFUNCTION
F13.180 - SEDATIVE, HYPNOTIC OR ANXIOLYTIC ABUSE W ANXIETY DISORDER
F13.18 - SEDATIVE, HYPNOTIC OR ANXIOLYTIC ABUSE W OTH DISORDERS
F13.159 - SEDATV/HYP/ANXIOLYTC ABUSE W PSYCHOTIC DISORDER, UNSP
F13.151 - SEDATV/HYP/ANXIOLYTC ABUSE W PSYCHOTIC DISORDER W HALLUCIN
F13.150 - SEDATV/HYP/ANXIOLYTC ABUSE W PSYCHOTIC DISORDER W DELUSIONS
F13.14 - SEDATIVE, HYPNOTIC OR ANXIOLYTIC ABUSE W MOOD DISORDER
F13.15 - SEDATIVE, HYPNOTIC OR ANXIOLYTIC ABUSE W PSYCHOTIC DISORDER
F13.129 - SEDATIVE, HYPNOTIC OR ANXIOLYTIC ABUSE W INTOXICATION, UNSP
F13.121 - SEDATV/HYP/ANXIOLYTC ABUSE W INTOXICATION DELIRIUM
F13.120 - SEDATV/HYP/ANXIOLYTC ABUSE W INTOXICATION, UNCOMPLICATED
F13.12 - SEDATIVE, HYPNOTIC OR ANXIOLYTIC ABUSE WITH INTOXICATION
F13.10 - SEDATIVE, HYPNOTIC OR ANXIOLYTIC ABUSE, UNCOMPLICATED
F13.1 - SEDATIVE, HYPNOTIC OR ANXIOLYTIC-RELATED ABUSE
F11.99 - OPIOID USE, UNSP WITH UNSPECIFIED OPIOID-INDUCED DISORDER
F11.988 - OPIOID USE, UNSPECIFIED WITH OTHER OPIOID-INDUCED DISORDER
F11.982 - OPIOID USE, UNSPECIFIED WITH OPIOID-INDUCED SLEEP DISORDER
F11.981 - OPIOID USE, UNSP WITH OPIOID-INDUCED SEXUAL DYSFUNCTION
F11.951 - OPIOID USE, UNSP W OPIOID-INDUC PSYCH DISORDER W HALLUCIN
F11.98 - OPIOID USE, UNSPECIFIED WITH OTH OPIOID-INDUCED DISORDER
F11.959 - OPIOID USE, UNSP W OPIOID-INDUCED PSYCHOTIC DISORDER, UNSP
F11.950 - OPIOID USE, UNSP W OPIOID-INDUC PSYCH DISORDER W DELUSIONS
F11.95 - OPIOID USE, UNSP WITH OPIOID-INDUCED PSYCHOTIC DISORDER
F11.94 - OPIOID USE, UNSPECIFIED WITH OPIOID-INDUCED MOOD DISORDER
F11.93 - OPIOID USE, UNSPECIFIED WITH WITHDRAWAL
F11.922 - OPIOID USE, UNSP W INTOXICATION WITH PERCEPTUAL DISTURBANCE
F11.929 - OPIOID USE, UNSPECIFIED WITH INTOXICATION, UNSPECIFIED
F11.92 - OPIOID USE, UNSPECIFIED WITH INTOXICATION
F11.920 - OPIOID USE, UNSPECIFIED WITH INTOXICATION, UNCOMPLICATED

F11.921 - OPIOID USE, UNSPECIFIED WITH INTOXICATION DELIRIUM
F11.90 - OPIOID USE, UNSPECIFIED, UNCOMPLICATED
F11.29 - OPIOID DEPENDENCE WITH UNSPECIFIED OPIOID-INDUCED DISORDER
F11.9 - OPIOID USE, UNSPECIFIED
F11.282 - OPIOID DEPENDENCE WITH OPIOID-INDUCED SLEEP DISORDER
F11.288 - OPIOID DEPENDENCE WITH OTHER OPIOID-INDUCED DISORDER
F11.281 - OPIOID DEPENDENCE WITH OPIOID-INDUCED SEXUAL DYSFUNCTION
F11.28 - OPIOID DEPENDENCE WITH OTHER OPIOID-INDUCED DISORDER
F11.251 - OPIOID DEPEND W OPIOID-INDUC PSYCHOTIC DISORDER W HALLUCIN
F11.259 - OPIOID DEPENDENCE W OPIOID-INDUCED PSYCHOTIC DISORDER, UNSP
F11.25 - OPIOID DEPENDENCE WITH OPIOID-INDUCED PSYCHOTIC DISORDER
F11.250 - OPIOID DEPEND W OPIOID-INDUC PSYCHOTIC DISORDER W DELUSIONS
F11.24 - OPIOID DEPENDENCE WITH OPIOID-INDUCED MOOD DISORDER
F11.23 - OPIOID DEPENDENCE WITH WITHDRAWAL
F11.229 - OPIOID DEPENDENCE WITH INTOXICATION, UNSPECIFIED
F11.222 - OPIOID DEPENDENCE W INTOXICATION WITH PERCEPTUAL DISTURBANCE
F11.221 - OPIOID DEPENDENCE WITH INTOXICATION DELIRIUM
F11.220 - OPIOID DEPENDENCE WITH INTOXICATION, UNCOMPLICATED
F11.20 - OPIOID DEPENDENCE, UNCOMPLICATED
F11.22 - OPIOID DEPENDENCE WITH INTOXICATION
F11.21 - OPIOID DEPENDENCE, IN REMISSION
F11.19 - OPIOID ABUSE WITH UNSPECIFIED OPIOID-INDUCED DISORDER
F11.2 - OPIOID DEPENDENCE
F11.188 - OPIOID ABUSE WITH OTHER OPIOID-INDUCED DISORDER
F11.182 - OPIOID ABUSE WITH OPIOID-INDUCED SLEEP DISORDER
F11.181 - OPIOID ABUSE WITH OPIOID-INDUCED SEXUAL DYSFUNCTION
F11.18 - OPIOID ABUSE WITH OTHER OPIOID-INDUCED DISORDER
F11.159 - OPIOID ABUSE WITH OPIOID-INDUCED PSYCHOTIC DISORDER, UNSP
F11.150 - OPIOID ABUSE W OPIOID-INDUCED PSYCHOTIC DISORDER W DELUSIONS
F11.151 - OPIOID ABUSE W OPIOID-INDUCED PSYCHOTIC DISORDER W HALLUCIN
F11.129 - OPIOID ABUSE WITH INTOXICATION, UNSPECIFIED
F11.14 - OPIOID ABUSE WITH OPIOID-INDUCED MOOD DISORDER
F11.15 - OPIOID ABUSE WITH OPIOID-INDUCED PSYCHOTIC DISORDER
F11.122 - OPIOID ABUSE WITH INTOXICATION WITH PERCEPTUAL DISTURBANCE
F11.121 - OPIOID ABUSE WITH INTOXICATION DELIRIUM
F11.12 - OPIOID ABUSE WITH INTOXICATION
F11.1 - OPIOID ABUSE
F11.120 - OPIOID ABUSE WITH INTOXICATION, UNCOMPLICATED
305.9 - DRUG ABUSE NEC & NOS
305.7 - AMPHETAMINE ABUSE
F11.10 - OPIOID ABUSE, UNCOMPLICATED

305.8 - ANTIDEPRESSANT ABUSE
305.6 - COCAINE ABUSE
305.5 - OPIOID ABUSE
305.4 - SEDATIVE/HYPNOTIC ABUSE
305.3 - HALLUCINOGEN ABUSE
304.9 - DRUG DEPENDENCE NOS
304.8 - COMB DRUG DEPENDENCE NEC
304.5 - HALLUCINOGEN DEPENDENCE
304.7 - OPIOID/OTHER DRUG DEP
304.2 - COCAINE DEPENDENCE
304.6 - DRUG DEPENDENCE NEC
304.1 - SEDATIVE/HYPNOTIC DEPEND
304.4 - AMPHETAMINE DEPENDENCE
648.34 - DRUG DEPENDENCE-POSTPART
648.32 - DRUG DEPENDEN-DEL W P/P
648.31 - DRUG DEPENDENCE-DELIVER
648.33 - DRUG DEPENDENCE-ANTEPART
305.93 - DRUG ABUSE NEC-IN REMISS
648.30 - DRUG DEPEND PREG-UNSPEC
305.92 - DRUG ABUSE NEC-EPISODIC
305.91 - DRUG ABUSE NEC-CONTIN
305.90 - DRUG ABUSE NEC-UNSPEC
305.83 - ANTIDEPRESS ABUSE-REMISS
305.81 - ANTIDEPRESS ABUSE-CONTIN
305.82 - ANTIDEPRESS ABUSE-EPISOD
305.80 - ANTIDEPRESS ABUSE-UNSPEC
305.72 - AMPHETAMINE ABUSE-EPISOD
305.73 - AMPHETAMINE ABUSE-REMISS
305.71 - AMPHETAMINE ABUSE-CONTIN
305.63 - COCAINE ABUSE-IN REMISS
305.70 - AMPHETAMINE ABUSE-UNSPEC
305.61 - COCAINE ABUSE-CONTINUOUS
305.62 - COCAINE ABUSE-EPISODIC
305.60 - COCAINE ABUSE-UNSPEC
305.53 - OPIOID ABUSE-IN REMISS
305.52 - OPIOID ABUSE-EPISODIC
305.51 - OPIOID ABUSE-CONTINUOUS
305.50 - OPIOID ABUSE-UNSPEC
305.42 - BARBITURATE ABUSE-EPISOD
305.43 - BARBITURATE ABUSE-REMISS
305.41 - BARBITURATE ABUSE-CONTIN

305.40 - BARBITURATE ABUSE-UNSPEC
305.33 - HALLUCINOG ABUSE-REMISS
305.31 - HALLUCINOG ABUSE-CONTIN
305.32 - HALLUCINOG ABUSE-EPISOD
305.30 - HALLUCINOG ABUSE-UNSPEC
304.92 - DRUG DEPEND NOS-EPISODIC
304.93 - DRUG DEPEND NOS-REMISS
304.90 - DRUG DEPEND NOS-UNSPEC
304.91 - DRUG DEPEND NOS-CONTIN
304.83 - COMB DRUG DEP NEC-REMISS
304.82 - COMB DRUG DEP NEC-EPISOD
304.81 - COMB DRUG DEP NEC-CONTIN
304.80 - COMB DRUG DEP NEC-UNSPEC
304.73 - OPIOID/OTHER DEP-REMISS
304.72 - OPIOID/OTHER DEP-EPISOD
304.70 - OPIOID/OTHER DEP-UNSPEC
304.71 - OPIOID/OTHER DEP-CONTIN
304.63 - DRUG DEPEND NEC-IN REM
304.61 - DRUG DEPEND NEC-CONTIN
304.60 - DRUG DEPEND NEC-UNSPEC
304.62 - DRUG DEPEND NEC-EPISODIC
304.53 - HALLUCINOGEN DEP-REMISS
304.51 - HALLUCINOGEN DEP-CONTIN
304.52 - HALLUCINOGEN DEP-EPISOD
304.50 - HALLUCINOGEN DEP-UNSPEC
304.43 - AMPHETAMIN DEPEND-REMISS
304.41 - AMPHETAMIN DEPEND-CONTIN
304.40 - AMPHETAMIN DEPEND-UNSPEC
304.42 - AMPHETAMIN DEPEND-EPISOD
304.23 - COCAINE DEPEND-REMISS
304.21 - COCAINE DEPEND-CONTIN
304.20 - COCAINE DEPEND-UNSPEC
304.22 - COCAINE DEPEND-EPISODIC
304.13 - BARBITURAT DEPEND-REMISS
304.12 - BARBITURAT DEPEND-EPISOD
304.03 - OPIOID DEPENDENCE-REMISS
304.11 - BARBITURAT DEPEND-CONTIN
304.10 - BARBITURAT DEPEND-UNSPEC
292.0 - DRUG WITHDRAWAL SYNDROME
304.01 - OPIOID DEPENDENCE-CONTIN
304.00 - OPIOID DEPENDENCE-UNSPEC

304.02 - OPIOID DEPENDENCE-EPISOD
F19.980 - OTH PSYCHOACTIVE SUBSTANCE USE, UNSP W ANXIETY DISORDER
F19.982 - OTH PSYCHOACTIVE SUBSTANCE USE, UNSP W SLEEP DISORDER
F19.981 - OTH PSYCHOACTIVE SUBSTANCE USE, UNSP W SEXUAL DYSFUNCTION
F19.98 - OTH PSYCHOACTIVE SUBSTANCE USE, UNSP W OTH DISORDERS
F19.97 - OTH PSYCHOACTIVE SUBSTANCE USE, UNSP W PERSISTING DEMENTIA
F19.96 - OTH PSYCHOACTV SUB USE, UNSP W PERSIST AMNESTIC DISORDER
F19.959 - OTH PSYCHOACTV SUBSTANCE USE, UNSP W PSYCH DISORDER, UNSP
F19.951 - OTH PSYCHOACTV SUB USE, UNSP W PSYCH DISORDER W HALLUCIN
F19.950 - OTH PSYCHOACTV SUB USE, UNSP W PSYCH DISORDER W DELUSIONS
F19.939 - OTHER PSYCHOACTIVE SUBSTANCE USE, UNSP WITH WITHDRAWAL, UNSP
F19.95 - OTH PSYCHOACTIVE SUBSTANCE USE, UNSP W PSYCHOTIC DISORDER
F19.94 - OTH PSYCHOACTIVE SUBSTANCE USE, UNSP W MOOD DISORDER
F19.932 - OTH PSYCHOACTV SUB USE, UNSP W W/DRAWAL W PERCEPTL DISTURB
F19.931 - OTH PSYCHOACTIVE SUBSTANCE USE, UNSP W WITHDRAWAL DELIRIUM
F19.93 - OTHER PSYCHOACTIVE SUBSTANCE USE, UNSP WITH WITHDRAWAL
F19.930 - OTH PSYCHOACTIVE SUBSTANCE USE, UNSP W WITHDRAWAL, UNCOMP
F19.929 - OTH PSYCHOACTIVE SUBSTANCE USE, UNSP WITH INTOXICATION, UNSP
F19.921 - OTH PSYCHOACTIVE SUBSTANCE USE, UNSP W INTOX W DELIRIUM
F19.922 - OTH PSYCHOACTV SUB USE, UNSP W INTOX W PERCEPTL DISTURB
F19.92 - OTHER PSYCHOACTIVE SUBSTANCE USE, UNSP WITH INTOXICATION
F19.920 - OTH PSYCHOACTIVE SUBSTANCE USE, UNSP W INTOXICATION, UNCOMP
F19.9 - OTHER PSYCHOACTIVE SUBSTANCE USE, UNSPECIFIED
F19.90 - OTHER PSYCHOACTIVE SUBSTANCE USE, UNSPECIFIED, UNCOMPLICATED
F19.29 - OTH PSYCHOACTIVE SUBSTANCE DEPENDENCE W UNSP DISORDER
F19.282 - OTH PSYCHOACTIVE SUBSTANCE DEPENDENCE W SLEEP DISORDER
F19.288 - OTH PSYCHOACTIVE SUBSTANCE DEPENDENCE W OTH DISORDER
F19.280 - OTH PSYCHOACTIVE SUBSTANCE DEPENDENCE W ANXIETY DISORDER
F19.281 - OTH PSYCHOACTIVE SUBSTANCE DEPENDENCE W SEXUAL DYSFUNCTION
F19.28 - OTH PSYCHOACTIVE SUBSTANCE DEPENDENCE W OTH DISORDERS
F19.251 - OTH PSYCHOACTV SUBSTANCE DEPEND W PSYCH DISORDER W HALLUCIN
F19.26 - OTH PSYCHOACTV SUBSTANCE DEPEND W PERSIST AMNESTIC DISORDER
F19.27 - OTH PSYCHOACTIVE SUBSTANCE DEPENDENCE W PERSISTING DEMENTIA
F19.250 - OTH PSYCHOACTV SUBSTANCE DEPEND W PSYCH DISORDER W DELUSIONS
F19.259 - OTH PSYCHOACTV SUBSTANCE DEPEND W PSYCHOTIC DISORDER, UNSP
F19.24 - OTH PSYCHOACTIVE SUBSTANCE DEPENDENCE W MOOD DISORDER
F19.25 - OTH PSYCHOACTIVE SUBSTANCE DEPENDENCE W PSYCHOTIC DISORDER
F19.239 - OTH PSYCHOACTIVE SUBSTANCE DEPENDENCE WITH WITHDRAWAL, UNSP
F19.231 - OTH PSYCHOACTIVE SUBSTANCE DEPENDENCE W WITHDRAWAL DELIRIUM
F19.232 - OTH PSYCHOACTV SUB DEPEND W W/DRAWAL W PERCEPTL DISTURB
F19.230 - OTH PSYCHOACTIVE SUBSTANCE DEPENDENCE W WITHDRAWAL, UNCOMP

F19.23 - OTHER PSYCHOACTIVE SUBSTANCE DEPENDENCE WITH WITHDRAWAL
F19.229 - OTH PSYCHOACTIVE SUBSTANCE DEPENDENCE W INTOXICATION, UNSP
F19.220 - OTH PSYCHOACTIVE SUBSTANCE DEPENDENCE W INTOXICATION, UNCOMP
F19.222 - OTH PSYCHOACTV SUBSTANCE DEPEND W INTOX W PERCEPTUAL DISTURB
F19.221 - OTH PSYCHOACTIVE SUBSTANCE DEPENDENCE W INTOX DELIRIUM
F19.20 - OTHER PSYCHOACTIVE SUBSTANCE DEPENDENCE, UNCOMPLICATED
F19.21 - OTHER PSYCHOACTIVE SUBSTANCE DEPENDENCE, IN REMISSION
F19.22 - OTHER PSYCHOACTIVE SUBSTANCE DEPENDENCE WITH INTOXICATION
F19.2 - OTHER PSYCHOACTIVE SUBSTANCE DEPENDENCE
F19.19 - OTH PSYCHOACTIVE SUBSTANCE ABUSE W UNSP DISORDER
F19.188 - OTH PSYCHOACTIVE SUBSTANCE ABUSE W OTH DISORDER
F19.181 - OTH PSYCHOACTIVE SUBSTANCE ABUSE W SEXUAL DYSFUNCTION
F19.180 - OTH PSYCHOACTIVE SUBSTANCE ABUSE W ANXIETY DISORDER
F19.182 - OTH PSYCHOACTIVE SUBSTANCE ABUSE W SLEEP DISORDER
F19.18 - OTH PSYCHOACTIVE SUBSTANCE ABUSE W OTH DISORDERS
F19.159 - OTH PSYCHOACTIVE SUBSTANCE ABUSE W PSYCHOTIC DISORDER, UNSP
F19.17 - OTH PSYCHOACTIVE SUBSTANCE ABUSE W PERSISTING DEMENTIA
F19.16 - OTH PSYCHOACTV SUBSTANCE ABUSE W PERSIST AMNESTIC DISORDER
F19.151 - OTH PSYCHOACTV SUBSTANCE ABUSE W PSYCH DISORDER W HALLUCIN
F19.15 - OTH PSYCHOACTIVE SUBSTANCE ABUSE W PSYCHOTIC DISORDER
F19.150 - OTH PSYCHOACTV SUBSTANCE ABUSE W PSYCH DISORDER W DELUSIONS
F19.14 - OTH PSYCHOACTIVE SUBSTANCE ABUSE W MOOD DISORDER
F19.129 - OTHER PSYCHOACTIVE SUBSTANCE ABUSE WITH INTOXICATION, UNSP
F19.121 - OTH PSYCHOACTIVE SUBSTANCE ABUSE WITH INTOXICATION DELIRIUM
F19.122 - OTH PSYCHOACTV SUBSTANCE ABUSE W INTOX W PERCEPTUAL DISTURB
F19.12 - OTHER PSYCHOACTIVE SUBSTANCE ABUSE WITH INTOXICATION
F19.120 - OTH PSYCHOACTIVE SUBSTANCE ABUSE W INTOXICATION, UNCOMP
F19.1 - OTHER PSYCHOACTIVE SUBSTANCE ABUSE
F19.10 - OTHER PSYCHOACTIVE SUBSTANCE ABUSE, UNCOMPLICATED
F16.988 - HALLUCINOGEN USE, UNSP W OTH HALLUCINOGEN-INDUCED DISORDER
F16.98 - HALLUCINOGEN USE, UNSP W OTH HALLUCINOGEN-INDUCED DISORDER
F16.99 - HALLUCINOGEN USE, UNSP W UNSP HALLUCINOGEN-INDUCED DISORDER
F16.983 - HALLUCIGN USE, UNSP W HALLUCIGN PERSIST PERCEPTION DISORDER
F16.980 - HALLUCINOGEN USE, UNSP W ANXIETY DISORDER
F16.950 - HALLUCINOGEN USE, UNSP W PSYCHOTIC DISORDER W DELUSIONS
F16.951 - HALLUCINOGEN USE, UNSP W PSYCHOTIC DISORDER W HALLUCINATIONS
F16.959 - HALLUCINOGEN USE, UNSP W PSYCHOTIC DISORDER, UNSP
F16.94 - HALLUCINOGEN USE, UNSP W HALLUCINOGEN-INDUCED MOOD DISORDER
F16.95 - HALLUCINOGEN USE, UNSP W PSYCHOTIC DISORDER
F16.929 - HALLUCINOGEN USE, UNSPECIFIED WITH INTOXICATION, UNSPECIFIED
F16.921 - HALLUCINOGEN USE, UNSP WITH INTOXICATION WITH DELIRIUM

F16.920 - HALLUCINOGEN USE, UNSP WITH INTOXICATION, UNCOMPLICATED
F16.92 - HALLUCINOGEN USE, UNSPECIFIED WITH INTOXICATION
F16.90 - HALLUCINOGEN USE, UNSPECIFIED, UNCOMPLICATED
F16.9 - HALLUCINOGEN USE, UNSPECIFIED
